# Natural Surfactant Saponin from Tissue of *Litsea glutinosa* and Its Alternative Sustainable Production

**DOI:** 10.3390/plants9111521

**Published:** 2020-11-09

**Authors:** Jiratchaya Wisetkomolmat, Ratchuporn Suksathan, Ratchadawan Puangpradab, Keawalin Kunasakdakul, Kittisak Jantanasakulwong, Pornchai Rachtanapun, Sarana Rose Sommano

**Affiliations:** 1 Interdisciplinary Program in Biotechnology, Graduate School, Chiang Mai University, Chiang Mai 50200, Thailand; jiratchaya_wis@cmu.ac.th; 2 Plant Bioactive Compound Laboratory, Department of Plant and Soil Sciences, Faculty of Agriculture, Chiang Mai University, Chiang Mai 50200, Thailand; 3 Research and Product Development, Department of Research and Conservation, Queen Sirikit Botanic Garden, The Botanical Garden Organisation, Chiang Mai 50180, Thailand; r_spanuchat@yahoo.com (R.S.); ratchadawanp@hotmail.com (R.P.); 4 Department of Entomology and Plant Pathology, Faculty of Agriculture, Chiang Mai University, Chiang Mai 50200, Thailand; kaewalin.k3@gmail.com; 5 Cluster of Agro Bio-Circular-Green Industry (Agro BCG), Chiang Mai University, Chiang Mai 50100, Thailand; jantanasakulwong.k@gmail.com (K.J.); pornchai.r@cmu.ac.th (P.R.); 6 Division of Packaging Technology, Faculty of Agro-Industry, Chiang Mai University, Chiang Mai 50100, Thailand; 7 Innovative Agriculture Research Centre, Faculty of Agriculture, Chiang Mai University, Chiang Mai 50200, Thailand

**Keywords:** compact mass spectrometry, cleansing properties, detergent plants, phytochemical, tissue culture

## Abstract

In this research, we assessed the detergency properties along with chemical characteristic of the surfactant extracts from the most frequently cited detergent plants in Northern Thailand, namely, *Sapindus rarak*, *Acacia concinna*, and *Litsea glutinosa*. Moreover, as to provide the sustainable option for production of such valuable ingredients, plant tissue culture (PTC) as alternative method for industrial metabolite cultivation was also proposed herein. The results illustrated that detergent plant extracts showed moderate in foaming and detergency abilities compared with those of synthetic surfactant. The phytochemical analysis illustrated the positive detection of saponins in *L. glutinosa* plant extracts. The highest callus formation was found in *L. glutinosa* explant cultured with MS medium supplemented with 2.0 mg/L Indole-3-acetic acid (IAA). The callus extract was chemical elucidated using chromatography, which illustrated the presence of saponin similar to those from the crude leaf and Quillaja saponin extracts. Compact mass spectrometry confirmed that the surfactant was of the steroidal diagnostic type.

## 1. Introduction

Surfactants are surface-active compounds with significant potentials for food-beverage, medicinal, and pharmaceutical industries. In surfactant manufacturing, they can be either chemically synthesized or found naturally in bio-based forms [1,2]. The first one is non-biodegradable, can be accumulated in the environment, and therefore is known to be toxic to living organisms [2]. Consequently, trends are moving towards finding the biological sources of the surfactants [1]. Biosurfactants can be derived directly from various natural sources including those of microbials, flora, and fauna [3,4,5]. Plant-based surfactants, such as saponins, have gained increasing attention as they are environmentally safe, less toxic, biodegradable, and renewable [6,7,8,9,10]. Structurally, saponins are amphipathic molecules consisted of triterpenoids, steroids, and glycosides [7,11,12]. Their aglycone parts are known as genin and sapogenin, which covalently bound to one or more polar molecules of sugar moieties [13,14,15,16]. Steroidal saponins such as diosgenin had been characterized and found in many plant samples [17,18,19]. Natural surfactants from plant resources are used mainly as detergents for fabric washing, hair, and body detergents with excellent cleansing properties [20]; for example, *Quillaja Saponaria* bark is used as a detergent for washing hair and clothes [21,22]. *Saponaria officinalis* is known as soap plant [13,23], and *Sapindus saponaria* is manufactured as soap and clothing washing detergent [24,25,26]. Some natural saponins have been traditionally used in folk medicines [4]. The drawback is that the high value phyto-intermediate raw materials are available naturally with limited abundance. Additionally, destructive harvesting may result in resource exhaustion and even nearly extinction from the nature [27]. More importantly, the degree functionality of active ingredient also varies upon environmental situations [28]. Due to these constraints, the sustainable production of raw material should be considered [27]. Plant tissue culture (PTC) is a potential alternative method to traditional plant propagation for retrieving natural products in the ways that multiplying and maintaining higher consistency and greater true-to-type raw material [29]. Moreover, this in vitro plant regeneration is also simple and time saving [28]. The underlaying advantage is that it can also help reducing the invasion of the natural resources and the destruction of biodiversity [30].

In the context of Northern Thai culture, three plant species namely *Sapindus rarak*, *Acacia concinna*, and *Litsea glutinosa* [31] are frequently mentioned as plants used in cleansing purposes. Among those, *L. glutinosa* was the most potential as the model plant for further conservation studies [32]. Belonging to the family Lauraceae, it also contains different kinds of secondary metabolites, such as alkaloids, tannins, sterols, and flavonoids, with good capability for medicinal purposes [33,34]. Leaf tissue of *L. glutinosa* comprises of numerous secondary metabolites such as megastigmane diglycoside, roseoside, pinoresinol 3-O-b-D-glucopyranoside [35], 2′-oxygenated flavone glycoside [36], and also natural saponin-surfactant with lists of proven active biofunctionalities [37,38]. It was also reported to be an endangered species or very rare in Asia, including in the Philippines, Bangladesh, and India [39]. However, the assessment of its detergency properties along with chemical characterization have not been reported in the literature. Thus, besides the ultimate aim to evaluate the cleaning properties of *L. glutinosa*, we also assessed the PTC as the alternative raw material propagation for extractable ingredients. The outcome of this study is therefore beneficial for ex situ conservation which inhibits the overexploitation of natural resource and the destruction of local forest to obtain natural material.

## 2. Results and Discussion

### 2.1. Foaming Ability, Stability, and Detergency Ability

Foaming ability, stability, and detergency ability of crude plant extracts are illustrated in Table 1. Foam produced by mechanical agitation is typically an unsteady thermodynamic system. Although foam generation has little to do with the cleansing ability of the detergent, it is an important criterion to evaluate detergent. Detergent can increase the ability to displace air from a liquid or solid surface [40,41]. By dissolving the crude detergent plant extracts, both of methanol or water extracts, in DI water at the concentration of 5 and 10% (*v/v*), the foaming regeneration of detergent plant extracts showed variable in the results after shaken. The highest foam was found in both types extracts from *S. rarak* (7.1 ± 0.10 cm) and the lowest was found in *L. glutinosa* (1.1 ± 0.06 cm). After 5 min, foam height decreased in all extracts. Chen et al. [40] explained that increase in foam height of detergent was due to the increasing in the concentrations and types of the detergents in aqueous solution. In the same study, foam produced by crude saponin extract of *Camellia oleifera* was less than that from the chemical synthesized products (SLS and Tween80) at 0.5% solution concentration. In the same way with the results from Yang et al. [41] who found that the crude saponin extract from *Sapindus mukorossi* showed lower ability of foaming than 0.5% SLS solution but similar to 0.5% Tween80 solution.

Different solvents used to extract the active ingredients could obviously play a role in the foaming properties. In our experiment, crude aqueous extract showed the highest content of foam compared to that of the methanol extract. The highest foam was found with 10% *v/v* methanol extract of *S. rarak* (7.1 cm). In the other hand, *L. glutinosa* extracts either by water or methanol gave low content of foam (1.1–1.5 cm). These are similar with Inalegwu and Sodipo [20] found that the crude aqueous extract of *Tephrosia vogelii* leaves illustrated higher foam height than that of the methanolic extract. Structural wise, saponins contain one or more sugar moieties attached to steroid or triterpenoid backbone. The number of sugar molecules could affect foaming characteristics with the lesser the sugar number, the lower foaming ability of the compound produced. It is also possible that some saponins possess no foam forming ability [7]. Böttcher and Drusch [42] also added that saponins from different plant resources illustrated variable in the interfacial properties, which could mask their true functionality. Not only that, crude extract may contain combination of various compounds such as protein that could contribute to the formation of foam [43].

Cleansing as described by the removal of dirt, soil, and grease, is the key important property of the common surfactants and detergency ability. This property is influenced by the content of surfactant ingredients in the extracted samples. From the result in Table 1, it was observed that methanol extracts gave much higher cleansing ability than that of the aqueous extract in all cases. Among the detergent plants tested, the cleansing ability of *A. concinna* was higher at either 5 or 10% than any others detergent plants. Here again, we noticed that such the ability increased with the increasing in the concentrations of all extracts except that of the aqueous extract of *L. glutinosa* that gave the moderate detergent ability (48.21 ± 1.71% detergent ability in 5% *v/v* water extract). Saponins have been proven to be the surfactive molecules that reduce surface tension and enhance cleansing ability [7,44]. Unsurprisingly, plants containing saponins are used as substitutes for soap with the hidden benefits as the treatment of skin lesions caused by fungi [45]. *A. concinna* pod has been traditionally used in India for bathing due to the formation of lather or foam in water. It also gave excellent cleansing actions on dirt similar to the commercial detergents or soaps [46]. Patel and Talathi [44] found that in herbal shampoo comprising of various aqueous plant extracts, the formulation with *A. concinna* extract had higher detergency ability (95.94%) than the extracts of *Aloe vera*, *S. mukorossi*, and *Phyllanthus emblica* because of the higher saponin content. There, nonetheless, is no report on the detergent ability of *L. glutinosa* elsewhere.

### 2.2. Chemical Properties

#### 2.2.1. Total Saponin Content

Total saponin content showed significant variations within the plant samples and ranged between 0.33–1.55 and 0.52–3.26 mg Diosgenin Equivalents (DE)/g extract, in crude water and crude methanol respectively. By using alcohol as the extracting solvent, *S. rarak* showed the highest amount of total saponin content (3.26 mg DE/g extract), followed by *A. concinna* (1.15 mg DE/g extract) and *L. glutinosa* (0.52 mg DE/g extract). In the same trend with the aqueous extracts, *S. rarak* showed the highest amount of total saponin content (1.88 mg DE/g extract), followed by *A. concinna* (0.52 mg DE/g extract) and *L. glutinosa* (0.33 mg DE/g extract). Notable that the detection of specific compound content in plant extract by using spectrophotometric method may cause an overestimation of the target compound content due to interferences with other components [47]. Moreover, choice of solvent used can also affect the total saponin content, and methanol is usually a much preferable choice for extraction of the compounds of higher polarity [17]. Preliminary phytochemical screening result from Khanpara et al. [48] reported that *A. concinna* fruit powder consisted of saponin (8.04%) and other compounds such as alkaloids, sugars, and flavonoids. Additionally, the study of Chavan et al. [49] revealed that fruits of *A. concinna* gave saponin as high as 10−11.5% and was known as “fruit for hair” that traditionally used as ingredient in traditional shampoo. Saponins exhibit surface properties such as low surface tension and shear visco-elasticity that possess mechanisms associated with foam and emulsion stabilization [50]. Saponin plants are indeed in need for detergent and cosmetic manufacturing. Nonetheless, the limitation of obtaining such the products from the natural resources is that the content is variable upon genotypes, geographical origin [51,52], and maturation state [45].

#### 2.2.2. Fourier Transform Infrared Spectroscopy (FTIR)

**Figure 1:**
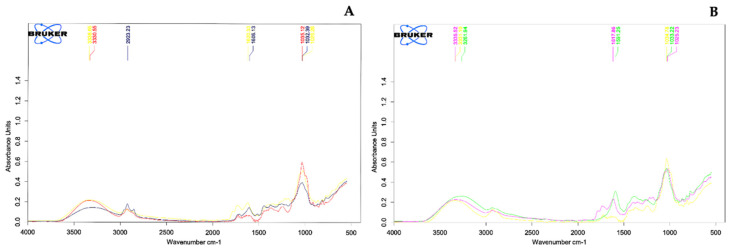
FTIR spectrum of crude detergent plants. (**A**) Crude methanol extracts; *A. concinna* (---) common absorption at 3338.65, 1620.33 and 1026.26 cm^−1^, *S. rarak* (---) 3330.55 and 1035.12 cm^−1^, *L. glutinosa* (---) 2923.23, 1606.13 and 1032.39 cm^−1^; (**B**) Crude water extracts; *A. concinna* (---) common absorption at 3335.52, 1617.86 and 1025.25 cm^−1^, *S. rarak* (---) 3331.10 and 1034.78 cm^−1^, *L. glutinosa* (---) 3261.94, 1591.25 and 1033.22 cm^−1^.

Identification of the functional groups can provide useful information on their mechanical and chemical properties [53]. Infrared spectroscopy is used to characterize the specific kind of bounds and the functional groups. The FTIR spectrum of crude plant extracts (prepared in methanol and water) are given in Figure 1A,B. The data of peak values and the possible functional groups of saponins from other literatures are presented in Supplementary Materials. FTIR spectrum of crude methanol or water extracts of the three detergent plants (*A. concinna*, *S. rarak*, and *L. glutinosa*) absorbed light at the wavenumber ranges of 1025–1620 and 2923–3338 cm^−1^. All detergent plant extracts showed characteristic infrared absorbance of the hydroxyl group (OH) at ~3338.65 cm^−1^ and ~3330.55 cm^−1^ in methanol extracts of *A. concinna* and *S. rarak* and the aqueous extracts gave the peaks at wave numbers at ~3335.52 cm^−1^ and ~3261.94 cm^−1^ of *A. concinna* and *L. glutinosa*, respectively. Carbon–hydrogen (C-H) absorption was found at 2923.23 cm^−1^ only in methanol extract of *L. glutinosa.* The C=C absorbance was observed at 1620.33 cm^−1^ (*A. concinna*) and 1606.13 cm^−1^ (*L. glutinosa*) in the water extracts but was not found in the both extracts of *S. rarak.* Whereas C-O-C absorbance varied from 1035.12 cm^−1^ (*S. rarak*) to 1026.26 cm^−1^ (*A. concinna*) in the methanol extracts and in the crude aqueous of *S. rarak* ~1034.78 cm^−1^ and 1033.22 cm^−1^ in *L. glutinosa*.

This result was similar with previous studies, as follows: Kareru et al. [54] reported characteristic infrared absorbance of phyto-based saponins of the hydroxyl group (OH) ranging from 3429 cm^−1^ to 3316 cm^−1^. Carbon–hydrogen (C-H) absorption ranged from 2931 cm^−1^ to 2931 cm^−1^ and oligosaccharide linkage absorptions to sapogenins (C-O-C) were at 1074 cm^−1^ and between 1045 cm^−1^ to 1046 cm^−1^. The study of Almutari and Ali [55] reported that saponins showed characteristic infrared absorbance of the hydroxyl group (OH) in soap nut extract, at 3407 cm^−1^ in the aqueous extract, 3419 cm^−1^ in the 95% ethanol extract, and from 3525 to 3281 cm^−1^ in the standard Quillaja saponin. The results of *Sapindus* extract in the study of Li et al. [56] also found that the stronger absorption of OH, CH_2_ and C-O-C in the sapindus extract spectrum, which similar to those of oleanolic acid spectrum (a standard of triterpenoid saponins). As *L. glutinosa* received much attention globally, and several studies were evaluated its chemical compositions and others biological activity [57,58,59] along with its potential detergency abilities reported herein. *L. glutinosa* was therefore chosen to study the alternative propagation method for bioactive compound induction.

### 2.3. In Vitro Plant Material Propagation

**Figure 2:**
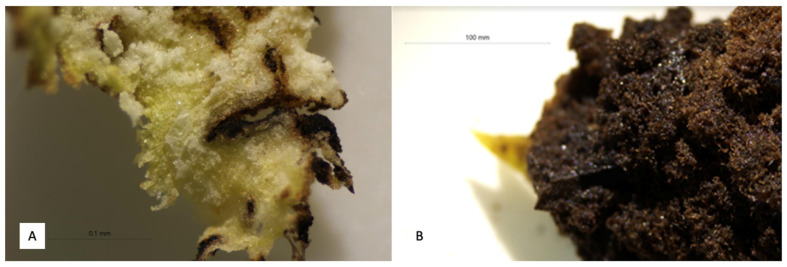
Callus formation; (**A**) Callus from treatment of 2,4-D; (**B**) Callus from treatment of IAA.

After six weeks of incubation, the callus induction from the tissue of *L. glutinosa* was observed. The highest callus formation was found in the explants cultured with Murashige and Skoog (MS) medium supplemented with 2.0 mg/L Indole-3-acetic acid (IAA). The greatest fresh weight was observed at 0.84 ± 0.47 g in IAA at concentration of 0.5 mg/L. Meanwhile the dry weight was the highest at 0.13 ± 0.03 g in 2,4-dichlorophenoxyacetic acid (2,4-D) at concentration of 2.0 mg/L (Table 2). Generally, the callus formed were completely friable with greenish and turned to brown colour after four weeks (Figure 2A,B). In addition, Wahyuni et al. [60] stated that the colour of callus becomes brown because they produced phenolic compounds and the variation in callus morphology is due to the explant variant, basic medium, growth regulators, and the biotic and abiotic supplements in the culture. In tissue culture, plant growth regulators play significant role in controlling plant growth and development [61]. Auxins are the major hormones responsible for stem elongation, vascular tissue differentiation, and cell expansion in plant developmental processes [62]. Similarly the cytokinins are required for cell division in a wide variety of plant tissue cultures [63]. During the micropropagation, factors influence the induction of callus are such as plants growth regulator, auxins, and cytokinins, and environmental condition, light, and temperature [64]. Therefore, in studying plant tissue culture for metabolites induction, at least two combination of hormone types are required. The study of callus induction for the production of saponin using different hormones and the combination thereof including 2,4-D and IAA combined with BAP and kinetin (KIN) [65,66]. The total saponin content (TSC) varied in the treatments range between 1.87 and 3.12 mg DE/g extract (Table 2). The callus was used in the evaluation of saponin content and chemical characterization thereafter.

### 2.4. High Performance Thin Layer Chromatography (HPTLC)

**Figure 3:**
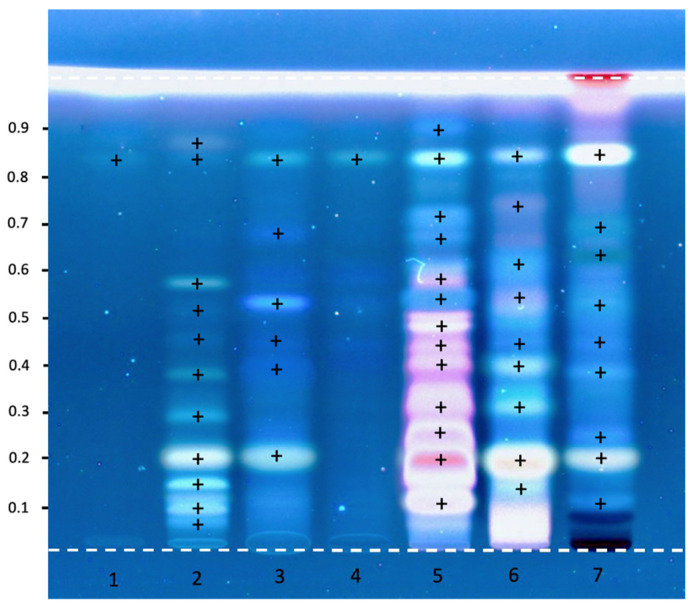
The HPTLC chromatogram of the crude methanolic of detergent plants after derivatized viewed under UV 366 nm. Track 1 = standard Diosgenin, 2 = standard Quillaja saponin, 3 and 4 = Callus of *Litsea* extracts, 5 = *Sapindus* extract, 6 = *Acacia* extract, 7 = *Litsea* extract. The symbol (+) indicate the presence of bands on each track.

HPTLC result in Figure 3 indicated that crude methanolic extracts of tissues of the detergent plants as well as the callus of *L. glutinosa* (LG callus) gave complex metabolites, while standard diosgenin gave a single band at the rate of flow (Rf) 0.84. For detection of saponins, the blue, violet, yellow, and green should be visible after derivatization at the visible light. They were best resolved at the wavelength UV 366 nm after derivatization [67,68]. Comparing the Rf values with the standard Quillaja saponin, the callus showed the spots at the similar Rfs of 0.2, 0. 39, 0.53 and 0.84, that were comparable with the methanolic extracts of *S. rarak*, *A. concinna*, and *L. glutinosa*, as shown in Figure 3. Other common peaks were also found in the methanolic extracts of detergent plants but were not observed in the standard diosgenin (Table 3).

In general, saponins from the same plant species can be found in the different forms of molecular structures [42]. Moreover, the variation of the structures could lead to the variety of physicochemical properties and biological activity [50]. The study of Senguttuvan and Subramaniam [68] reported the HPTLC fingerprints of various metabolites in *Hypochaeris radicata* L. and saponins can detected by using the mobile phase consisting of chloroform: glacial acetic acid: methanol: water. The methanolic plant extracts displayed different Rf pattern and the root extract attained the greater number of saponins than the leaf extracts. Further, study of Das et al. [69] showed the variable in the Rf peaks of HPTLC chromatograms of *Diplazium esculentum* Retz. and suggested that the ethanolic extract could yield variety of phenolics, flavonoids, and saponins than those found in the aqueous extract. The type of saponin in the extract was then confirmed by mass spectrometry.

### 2.5. Compact Mass Spectrometry (CMS)

**Figure 4:**
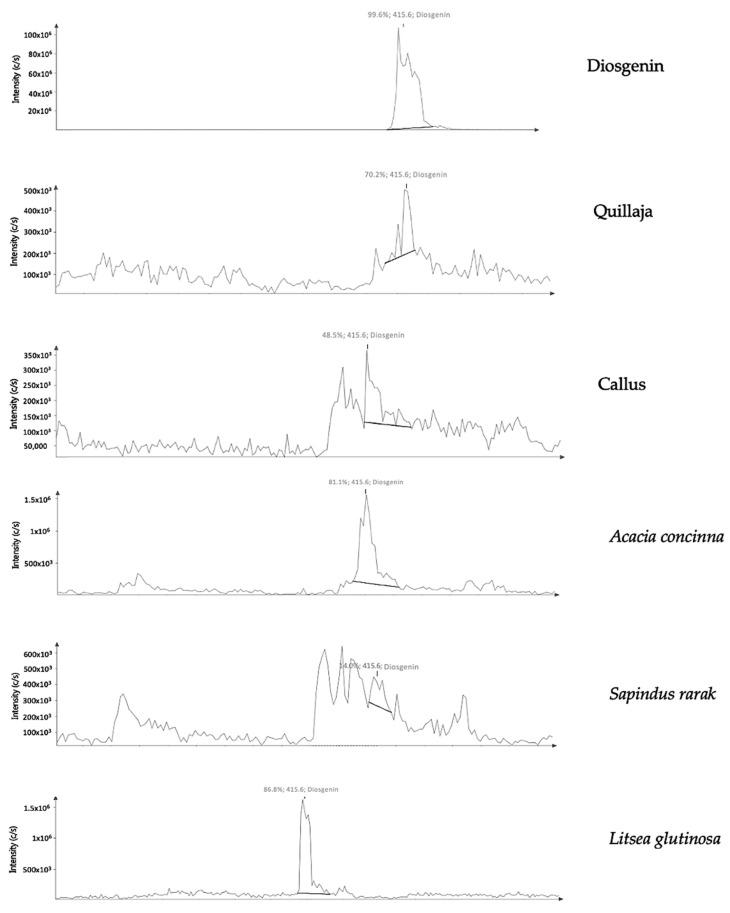
Mass spectra of compound likes diosgenin detected the ion current abundance of *m/z* 415.6 in methanolic extract of detergent plant, callus compared with standard contained saponin.

**Scheme 1:**
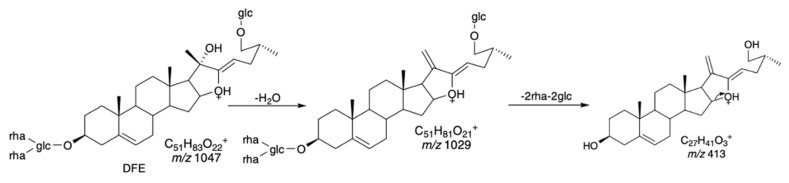
The proposed of fragmentation patterns of DFE.

Mass spectroscopy (MS) is the systematics study of mass spectrum with or without fragmentation which is characterized by a relationship between the mass of a given ion and the number of elementary charges that it carries or mass-to-charge ratios (*m/z*) and relative abundances [70]. Mass spectra of any given substances are different in fragmentation patterns. These fragmentation patterns are useful to determine the molar weight and structural information of the unknown molecule [71]. The mass spectra of the methanolic detergent plant extracts and callus of *L. glutinosa* gave the identical peak with the *m/z* 415.6 in positive ion mode with the standard diosgenin (Table 4). Among the vast array of *m/z* data of the samples, many peaks were also found in common (Figure 4). In the report of Li et al. [72] elucidated that the *m*/*z* value of 415.3217 was according to the presence protonated aglycone which was the fragmentation of steroidal saponins, 26-*O-β*-D-glucopyranosyl-3*β*,20*α*,26-triol-25(R)-Δ-dienofurostan-3-*O*-*α*-L-rhamnopyranosyl(1→2)-[*α*-L-rhamnopyranosyl(1→4)]-*β*-D-glucopyranoside (DFE) (Scheme 1). In another study, fragment of aglycone with the *m*/*z* value of 415 was detected in the terrestrinin S (or pseudosapogenins of Type IV) which confirmed diagnostic ions of steroidal saponin [73]. Thus, the CMS *m/z* results of crude saponin extracts from all detergent plants along with the callus produced by *L. glutinosa* tissue in our study could be structurally characterized as the steroidal diagnostic type.

## 3. Materials and Methods

### 3.1. Chemicals and Standards

Diosgenin (with molecular weight of 414.5 g/mol and purity of ≥93%) was purchased from Sigma-Aldrich (Steinheim, Germany). Saponin from Quillaja Bark pure, 10–14% sapogenin content, was product of PanReac AppliChem (Darmstadt, Germany). The methanol, ethanol, and sulfuric acid (98%) were products of RCI Labscan (Bangkok, Thailand). Vanillin (99%) was product of Sigma-Aldrich (Steinheim, Germany). Sodium Lauryl Sulfate (SLS) was product of KEMAUS (New South Wales, Australia).

### 3.2. Collection and Authentication of Plant Materials

All plant materials used in this study were collected from the forest reserve restored by the Huai Hong Khrai Royal Development Study Centre (HHKC) during the fields study reported elsewhere [32]. Plant species confirmation was done by comparison the specimen with those deposited at plant collection library of the ethnobotanical laboratory, Department of Biology, Faculty of Science, Chiang Mai University (CMU). The specimens were deposited at Plant Bioactive Compound Laboratory Herbarium (BACH), Faculty of Agriculture with the voucher number JW009-011. The utilized parts, including: pericarp of *Sapindus rarak* (family Sapindaceae), fruit of *Acacia concinna* (family Fabaceae) and Leaves of *Litsea glutinosa* (family Lauraceae) were used in this study. The plant parts were separated, cleaned and dried at 40 °C for 72 h in hot-air oven (WGLL-125BE, Huanghua faithful, Hebei, China). Thereafter, they were grounded into powder using a hand-held grinder (Philips, The Netherlands) at speed level 1 prior to solvent extraction and stored in an air-tight container for further studies. To screen saponin content in plant materials, dry sample powder 0.05 g was suspended in 80% methanol (*v/v*) and extracted using ultrasonication. Unless otherwise stated, the supernatant was used to in colorimetric method for saponin determination later described.

### 3.3. Determination of Detergent Properties

Unless stated otherwise, 10 g of dried material was extracted with 100 mL of either deionized water (DIH_2_O) or 80% methanol at room temperature (25 ± 2 °C) according to the method of Inalegwu and Sodipo [20]. The filtrate was concentrated using automate rotary evaporator under reduced pressure at 40 °C until dried. To evaluate the physiochemical properties of *L. glutinosa*, *A. concinna*, and *S. rarak* were selected to compare their properties based on the ethnobotanical report from our previous study. The detergent properties determined were as followed;

#### 3.3.1. Foaming Ability and Stability

Foaming ability was evaluated according to the methods of Chen et al. [40]; Inalegwu and Sodipo [20] and Pradhan and Bhattacharyya [74] with slight modifications. The extracts were dissolved in water at the concentrations of 5 and 10% (*v/v*). The solution was then vigorously shaken for 1 min by hand in a 10 mL measuring cylinder and the foam height was measured at room temperature. Foam stability and foam height after 30 s and 5 min were also recorded. The measurements were repeated three times from separate set of samples and the values were averaged.

#### 3.3.2. Detergency Ability

The detergent abilities of the crude extract were evaluated following the method of Pradhan and Bhattacharyya [74] with some modifications. The test was conducted using 5 × 5 cm cotton cloth pieces were cleaned with a 5% Sodium Lauryl Sulfate (SLS) solution, then dried. The samples were suspended in artificial sebum (consisting of 1 g coconut oil and 1 g paraffin wax in 100 mL hexane solution) and the mixture was shaken for 15 min. Then samples were removed and left to dryness at room temperature. In the next step, oil-coated materials were divided into two equal portions, the first part was washed with 10 mL of the 5 and 10% crude extracts (both methanol and water fractions) and the second group was cleaned with deionized water (control). After drying, the resided sebum on samples was extracted with 20 mL hexane for 15 min and the materials were removed. After evaporated off to dryness, the sebum content was weighed. Finally, the percentage of detergency ability in both cases was calculated using the following equation;

Detergency Ability = 100 − (T/C × 100)(1)

In which, DA is the percentage of detergency ability. C is the weight of sebum in the controls and T is the weight of sebum in the test sample.

### 3.4. Chemical Analyses

#### 3.4.1. Determination Saponin Contents

The method of Makkar [75] with slight modification was used to determine the total saponin content in plant materials. Briefly, 50 µL of plant extract was mixed with 2.5 mL sulfuric acid 72% (*v/v*) and 0.1 mL, 8% vanillin solution in ethanol. The mixtures were incubated in a water bath at 60 °C for 10 min and then cooled in cold water. The absorbance of sample was measured at 544 nm using SPECTROstar Nano Microplate Reader (BMG LABTECH, Ortenberg, Germany). Diosgenin was used as the reference standard with saponin content expressed as mg Diosgenin Equivalents (DE) per gram extract (mg DE/g extract). The final quantification of the phytochemicals is the value of mean ± SD of three measurements.

#### 3.4.2. Fourier Transform Infrared Spectroscopy (FTIR)

To identify the presence of saponin functional groups, the FTIR spectra of the extracts were collected by a Bruker model ALPHA II, diamond ATR (Hamburg, Germany) and operating at the basic of 500–4000 wavenumber for averaging 47 scans per spectrum. For the analysis of the different chemical bonds in crude extracts, both methanol and water extracts were used for the FTIR measurement [42].

### 3.5. In Vitro Plant Material Propagation

The lateral bud of plant material was used in this study. For surface sterilization procedures, the explants were soaked in filtered distilled water followed by 95% ethanol for 5 min. Thereafter, the explants were soaked in 25% of sodium hypochlorite solution added with 2–3 drops of tween 20 (Sigma, St. Louis, MO, USA) for 20 min. The explants were then thoroughly rinsed with sterile distilled water to remove any traces of remaining detergents [76].

The explants were inoculated in MS culture medium. The media consisted of 30 g/L sucrose and 7 g/L agar and the pH was adjusted to be within 5.68 after the addition of plant growth regulators. Different concentration, 0.5, 1.0, and 2.0 mg/L of two auxin (2,4-dichlorophenoxyacetic acid (2,4-D) and Indole-3-acetic acid (IAA)) and two cytokinin (6-Benzylaminopurine (BA) and Kinetin (KIN)) were used in the study. The cultures were incubated at 25 ± 2 °C.

The observation of callus was done on a weekly basis. At the end of four weeks, the formed callus was isolated from the explants. The data for callus induction in each treatment was recorded in which the morphology, fresh, and dry weights (g) [76]. The percentage of callus initiation was calculated [30]. Thereafter, the content of total saponin was evaluated and characterization on HPTLC and CMS.

### 3.6. High Performance Thin Layer Chromatography (HPTLC)

HPTLC finger printing study was carried out according to the methods of Avula et al. [77]; Karthika et al. [67]; and Senguttuvan and Subramaniam [68]. The HPTLC system comprising of Linomat 5 automatic applicator, a twin trough plate development chamber, Camag TLC scanner 3 and winCATS 4 software version 2.5.18262.1 (CAMAG, Muttenz, Switzerland) were used. The methanolic extract of the callus, detergent plants (10 μL) and 3 μL of standards (Diosgenin and *Quillaja* saponins) were spotted separately in form of bands having band width of 5 mm on glass plates (Merck, Darmstadt, Germany) with silica gel 60 F_254_ (20 × 10 cm), using a Hamilton syringe with Linomat 5 applicator attached to CAMAG HPTLC system. The plate was accommodated with 7 tracks and all samples were applied according to the following settings: 8 mm from the bottom of the plate, band width 8 mm; application volume 3–10 µL. All remaining measurement parameters were left at default settings. Chamber saturation was done using 20 × 10 cm Whatman filter paper for 20 min. Development solvent was the lower layer of chloroform: glacial acetic acid: methanol: H_2_O (6.4:3.2:1.2:0.8) till 80 mm from the lower edge of the plate. Then chromatogram was developed in the twin trough glass chamber (20 × 10 cm) presaturated with the mobile phase. Developed plates were immersed in Anisaldehyde sulfuric acid reagent, and dried on heat plate for 5 min at 100 °C. The dried plates were kept in a photo documentation chamber and captured the images in visible light, UV 366 nm and UV 254 nm.

### 3.7. Compact Mass Spectrometry (CMS)

The experiment was performed using the expression Compact Mass Spectrometer and Atmospheric Solids Analysis Probe (ASAP) from Advion, Inc. (Ithaca, NY, USA) to measure the presence of saponin compounds contained in the complex matrix including methanol extracts of detergent plants, callus of selected detergent plant in comparison with the standards following the standard protocol of Perez-Hurtado et al. [78]. The instrument was operated in positive ionization mode. All the spectra were acquired within the range of *m/z* 400–430.

### 3.8. Statistical Analysis

The extractions of phytochemicals and analyses were of three separate of each sample. The data were subjected to Analysis of Variance (ANOVA) and comparison between the mean values of treatment were confirmed by Duncan’s Multiple Range test at the 95% level of significance. A completely randomized design (CRD) with 25 replicates was performed to determine the effect of plant growth regulator on callus induction. The data were presented as mean ± SD. The means comparison and statistical analysis were done using SPSS 17.0 software [76].

## 4. Conclusions

The recent study assessed the detergency properties along with chemical confirmation of the most cited detergent plant in northern Thailand and its alternative propagation for active ingredient production using plant tissue culture. The results from detergent abilities, physiochemical properties, and phytochemical analysis showed the positive detection of saponins present in *L. glutinosa* plant extracts. Using IAA at 2.0 mg/L concentration in MS media could produce callus that yielded metabolites of different forms. Further study can investigate in-depth on derivatives of saponin, identification, and structure elucidation.

## Figures and Tables

**Table 1:** Detergent properties of crude detergent plant extracts.

Extracts	Type of Extracts	Concentration (% *v/v*)	Foam Height (cm)		Detergency Ability (%)
			30 s	5 min	
Control	SLS	0.5	7.93 ± 0.06 ^a^	7.03 ± 0.06 ^a^	88.98 ± 1.92 ^a^
*Acacia concinna*	Methanol	5	2.30 ± 0.10 ^g^	1.03 ± 0.21 ^f^	71.52 ± 2.70 ^d^
	Methanol	10	3.10 ± 0.10 ^f^	1.13 ± 0.12 ^f^	82.43 ± 1.33 ^b^
	Water	5	5.43 ± 0.21 ^e^	4.47 ± 0.15 ^e^	59.65 ± 2.67 ^fg^
	Water	10	6.10 ± 0.20 ^d^	5.13 ± 0.15 ^d^	78.42 ± 1.10 ^c^
*Sapindus rarak*	Methanol	5	5.93 ± 0.12 ^d^	5.30 ± 0.10 ^d^	66.60 ± 1.69 ^f^
	Methanol	10	7.10 ± 0.10 ^b^	6.57 ± 0.25 ^b^	74.44 ± 1.90 ^d^
	Water	5	6.87 ± 0.06 ^c^	5.77 ± 0.15 ^c^	57.38 ± 0.66 ^fg^
	Water	10	7.13 ± 0.15 ^b^	6.74 ± 0.17 ^b^	64.29 ± 3.41 ^f^
*Litsea glutinosa*	Methanol	5	1.13 ± 0.06 ^i^	0.60 ± 0.10 ^g^	54.26 ± 2.96 ^g^
	Methanol	10	1.13 ± 0.15 ^i^	0.60 ± 0.10 ^g^	65.69 ± 2.81 ^f^
	Water	5	1.37 ± 0.12 ^h^	0.47 ± 0.06 ^g^	48.21 ± 1.71 ^h^
	Water	10	1.48 ± 0.08 ^h^	0.50 ± 0.10 ^g^	56.06 ± 1.69 ^fg^

Data are expressed as the mean ± SD of three independent experiments; means with the superscription letters (a–i) are significantly different at *p* ≤ 0.05.

**Table 2:** Callus induction in *L. glutinosa* from stem cultured on MS medium treated with various concentrations of auxins (2,4-D and IAA) and 2 cytokinin (kinetin (KIN) and BA) hormones after four weeks of cultured.

Hormone	Concentration (mg/L)	% Callus Induction	Fresh Weight (g)	Dry Weight (g)	TSC
2,4-D	0.5	70 ± 14.14 ^c^	0.53 ± 0.24 ^b^	0.03 ± 0.01 ^e^	1.91 ± 0.03 ^de^
	1.0	76 ± 16.97 ^bc^	0.62 ± 0.25 ^b^	0.09 ± 0.05 ^b^	2.01 ± 0.04 ^d^
	2.0	48 ± 5.66 ^de^	0.61 ± 0.13 ^b^	0.13 ± 0.03 ^a^	2.00 ± 0.16 ^d^
IAA	0.5	90 ± 8.49 ^ab^	0.84 ± 0.47 ^a^	0.07 ± 0.03 ^c^	2.56 ± 0.01 ^c^
	1.0	92 ± 5.66 ^ab^	0.56 ± 0.36 ^b^	0.07 ± 0.05 ^c^	2.73 ± 0.07 ^b^
	2.0	100 ± 0.00 ^a^	0.62 ± 0.29 ^b^	0.05 ± 0.03 ^d^	3.12 ± 0.10 ^a^
KIN	0.5	34 ± 8.49 ^ef^	-	-	-
	1.0	32 ± 5.66 ^ef^	-	-	-
	2.0	20 ± 5.66 ^f^	-	-	-
BA	0.5	64 ± 5.66 ^cd^	0.06 ± 0.03 ^c^	0.01 ± 0.00 ^f^	1.85 ± 0.01 ^e^
	1.0	66 ± 2.83 ^cd^	0.06 ± 0.03 ^c^	0.01 ± 0.00 ^f^	1.79 ± 0.08 ^e^
	2.0	38 ± 2.83 ^ef^	0.05 ± 0.01 ^c^	0.01 ± 0.00 ^f^	2.68 ± 0.04 ^bc^

Data are expressed as the mean ± SD; means with the superscription letters (a–f) are significantly different at *p* ≤ 0.05.

**Table 3:** The rate of flow (Rf) values of the methanolic detergent plant extracts compared with saponin standard from High Performance Thin Layer Chromatography (HPTLC).

Spot No.	Diosgenin	Quillaja	*S. rarak*	*A. concinna*	*L. glutinosa*	LG Callus
1		0.07				
2		0.10	0.12		0.12	
3		0.15		0.14		
4		0.20	0.19	0.19	0.20	0.21
5			0.26			
6		0.29	0.32	0.32		
7		0.38	0.40	0.40	0.38	0.39
8		0.46	0.44	0.44	0.45	0.45
9			0.48			
10		0.52	0.54	0.54	0.53	0.53
11		0.58	0.59	0.62	0.64	
12			0.68			0.69
13			0.72	0.74	0.70	
14	0.84	0.84	0.84	0.84	0.84	0.84
15		0.87	0.90			

**Table 4:** Mass spectra of detergent plants methanolic extracts.

Label	Maximum Intensity (c/s)	% Peak Area	Base Peak Mass (*m/z*)
Diosgenin	1.1 × 10^8^	99.6	415.6
Quillaja	3.1 × 10^5^	70.2	415.6
*A. concinna*	1.4 × 10^6^	81.1	415.6
*S. rarak*	1.7 × 10^5^	14	415.6
*L. glutinosa*	1.5 × 10^6^	86.8	415.6
Callus	2.4 × 10^5^	48.5	415.6

## Supplementary Materials

The following is available online at https://www.mdpi.com/2223-7747/9/11/1521/s1, Table S1: FTIR spectra of functional groups of saponins.
