# Supplementary material for: Natural Surfactant Saponin from Tissue of Litsea glutinosa and Its Alternative Sustainable Production
Source: Plants (Basel). 2020 Nov 9;9(11):1521. doi: 10.3390/plants9111521 (PMC7695332; doi:10.3390/plants9111521)
Supplement: Supplementary file 1 [file plants-09-01521-s001.pdf]

Table S1. FTIR spectra of functional groups of saponins.

FTIR Spectrum	Functional Groups		References
3429-3316	Hydroxyl group (saponin)	-OH	[53,54]
3400	C-OH stretching (saponin)		[42]
3200-2800	sp^2 and sp^3 -C-H bond stretching (saponin)		[42]
2929-2920		C-H	[53,54]
2926-2855	Indicated more CH_2 than CH_3		[56]
1740-1736		C=O	[54]
1724-1701	Carbonyl group	C=O	[53]
1729-1697		C=O	[56]
1730-1700	C=O (the stretch of the carbonyl bonds)		[42]
1619-1651		C=C	[54]
1639	C=O stretching		[8]
1637-1632		C=C	[56]
1600	C=C (saponin)		[42]
1256-1062	C-O-C and carboxylic ester groups	C-O-C	[8, 55]
1034-1074	Oligosaccharide linkage absorptions to sapogenins	C-O-C	[54,55]
